# Research advance of natural products in tumor immunotherapy

**DOI:** 10.3389/fimmu.2022.972345

**Published:** 2022-09-02

**Authors:** Jin-Ling Huo, Wen-Jia Fu, Zheng-Han Liu, Nan Lu, Xiang-Qian Jia, Zhang-Suo Liu

**Affiliations:** ^1^ Department of Integrated Traditional and Western Nephrology, the First Affiliated Hospital of Zhengzhou University, Research Institute of Nephrology, Zhengzhou University, Henan Province Research Center For Kidney Disease, Key Laboratory of Precision Diagnosis and Treatment for Chronic Kidney Disease in Henan Province, Zhengzhou, China; ^2^ Engineering Research Center of Agricultural Microbiology Technology, Ministry of Education, Heilongjiang University, Harbin, China; ^3^ Heilongjiang Provincial Key Laboratory of Ecological Restoration and Resource Utilization for Cold Region, School of Life Sciences, Heilongjiang University, Harbin, China; ^4^ School of Mechanical and Aerospace Engineering, Nanyang Technological University, Singapore, Singapore

**Keywords:** tumor immunotherapy, immune cells, natural products, active chemicals, tumor microenvironment

## Abstract

Cancer immunotherapy has emerged as a novel anti-tumor treatment. Despite significant breakthroughs, cancer immunotherapy remains focused on several types of tumors that are sensitive to the immune system. Therefore, effective strategies to expand its indications and improve its efficacy become key factors for the further development of cancer immunotherapy. In recent decades, the anticancer activities of natural products are reported to have this effect on cancer immunotherapy. And the mechanism is largely attributed to the remodeling of the tumor immunosuppressive microenvironment. The compelling data highlight that natural products offer an alternative method option to improve immune function in the tumor microenvironment (TME). Currently, more attention is being paid to the discovery of new potential modulators of tumor immunotherapy from natural products. In this review, we describe current advances in employing natural products and natural small-molecule drugs targeting immune cells to avoid tumor immune escape, which may bring some insight for guiding tumor treatment.

## Introduction

The immune system is a sophisticated integrated network consisted of various immune cells, organs, and soluble mediators that developed to protect the biosome against outside assault which threatens the integrity of biosome (1). The immune system’s critical role in host defense is best seen when things go wrong; underactivity leads to severe infections and tumors, while abnormal activity leads to allergy and autoimmune illnesses, especially tumor escape. Immune cells are an important part of the immune system, including lymphocytes, dendritic cells (DCs), monocytes/macrophages, natural killer (NK) cells, and so on (2). It is now clear that in addition to cancer cells, the tumor microenvironment (TME) contains a repertoire of endothelial cells, stromal cells and immune cells (3). In contrast to traditional chemotherapy, immunotherapy focuses on the specific recognition and attack of cancer cells using immune cells inside and outside of TME (4). Thus, immunotherapy approaches are theoretically shown to have higher specificity and lower side effects.

Cancer immunotherapy is defined that regulates immunological response through activating the organism’s immune defense system to suppress and prevent tumor growth (5). Now, a variety of cancer vaccines, immune-checkpoint inhibitors and adoptive immune-cell immunotherapies for anti-cancer treatments are approved by the US Food and Drug Administration (FDA) (6–8). Despite significant breakthroughs, cancer immunotherapy remains focused on several types of tumors that are sensitive to the immune system. The main reason for this limitation is the immunosuppressive microenvironment within the tumor site, which effectively blunts cancer immunotherapy (9). Therefore, effective strategies that can reverse and reshape the complex immunosuppressive microenvironment within tumors are a key factor in expanding the indications for cancer immunotherapy.

Natural products used to treat human diseases can date back over 3000 years and contain several active components found in medicinal plants. Furthermore, mangy natural products were verified and documented in several publications in ancient China. Numerous studies currently have demonstrated that natural products play an important role in the development of new anti-cancer drugs and lead compounds due to their wide range of sources, low cost, structural diversity, diverse biological activities and low adverse effects (10, 11). Several well-known natural products, including polysaccharides (e.g., astragalus polysaccharides, shiitake polysaccharides), alkaloids (e.g., matrine, berberine), saponins (e.g., ginsenoside, total saponin of acanthopanax bark), flavonoids (e.g., baicalin, apigenin), and terpenoids (e.g., artemisinin, paclitaxel) have potential anti-tumor immunomodulatory effects (12) (**Table 1**). In addition, natural products have achieved great success in effectively expanding the indications and improving the efficacy of various types of cancer immunotherapy, such as immune checkpoint inhibitors, cancer vaccines, and adoptive immune cell transfer therapy (13–15). In this paper, we describe the immunomodulatory effects of natural products, as well as the underlying processes of the immune response activation in TME (16–18).

**Table 1:** Representative natural products with immuno-tumor therapeutic effects.

Category	Natural product	Source	Mechanism
Polysaccharides	Ascophyllan	Ascophyllum nodosum	Increasing MHC I, MHC II and pro-inflammatory levels of cytokines; ultimately inducing activation of DCs and antigen-specific immune responses
	Ganodema polysaccharides	Ganodema	Down-regulate the expression of PD-1 and PD-L1 through STAT3 pathway
	CMPB90-1	Cordyceps militaris	Down-regulate the expression of PD-L1 through NF-kB pathway
	Fucoidan	Ascophyllum nodosum	Promotes the growth of human peripheral blood DC
Alkaloids	Chloroquine	Cinchona bar	Increasing the lysosomal pH of TAM, mediating Ca2+ release and activating TFEB
	Tryptanthrin-5c	Polygonum tinctorium and Isatis tinctoria	nhibits the activity of IDO and treg accumulation
	5-Br-brassinin	Cruciferous sp.	Inhibiting the activity of IDO1 and mediating tumor regression when combined with chemotherapeutic drugs in MMTV-Neu mice
Saponins	QS-21	Quillaja saponaria Molin	Enhance the anti-cancer effect of cancer vaccines
	Sapogenin	Panax ginseng	Down-regulation of PD-1 and PD-L1 expression through the STAT3 pathway
	Diosgenin	Acacia concinna	Enhancing the anti-cancer effects of anti-PD-1 antibodies
Flavonoids	Hesperidin	Orange peel	Downregulation of PD-L1 expression *via* NF-kB pathway
	Baicalein	Scutellaria	Downregulation of PD-L1 expression *via* JAKSTAT pathway
	Procyanidin	Fruits	Enhancing the anti-cancer effects of cancer peptide vaccines
Terpenoids	Artemisinin	Artemisia annua	Inhibiting the proliferation of MDSCs and Tregs, and promoting the proliferation of CD4 + T and CD8 + T cells
	Ginsenoside Rk1	Black ginseng	Downregulation of PD-L1 by inhibiting NF-κB signaling
	Ingenol-3,20-dibenzoate	Euphorbia esula L	Activating PKC, promoting IFN-γ secretion and degranulation, and ultimately increasing NK cell cytotoxicity

## Immune modulation of natural products to immune cells

**Figure 1:**
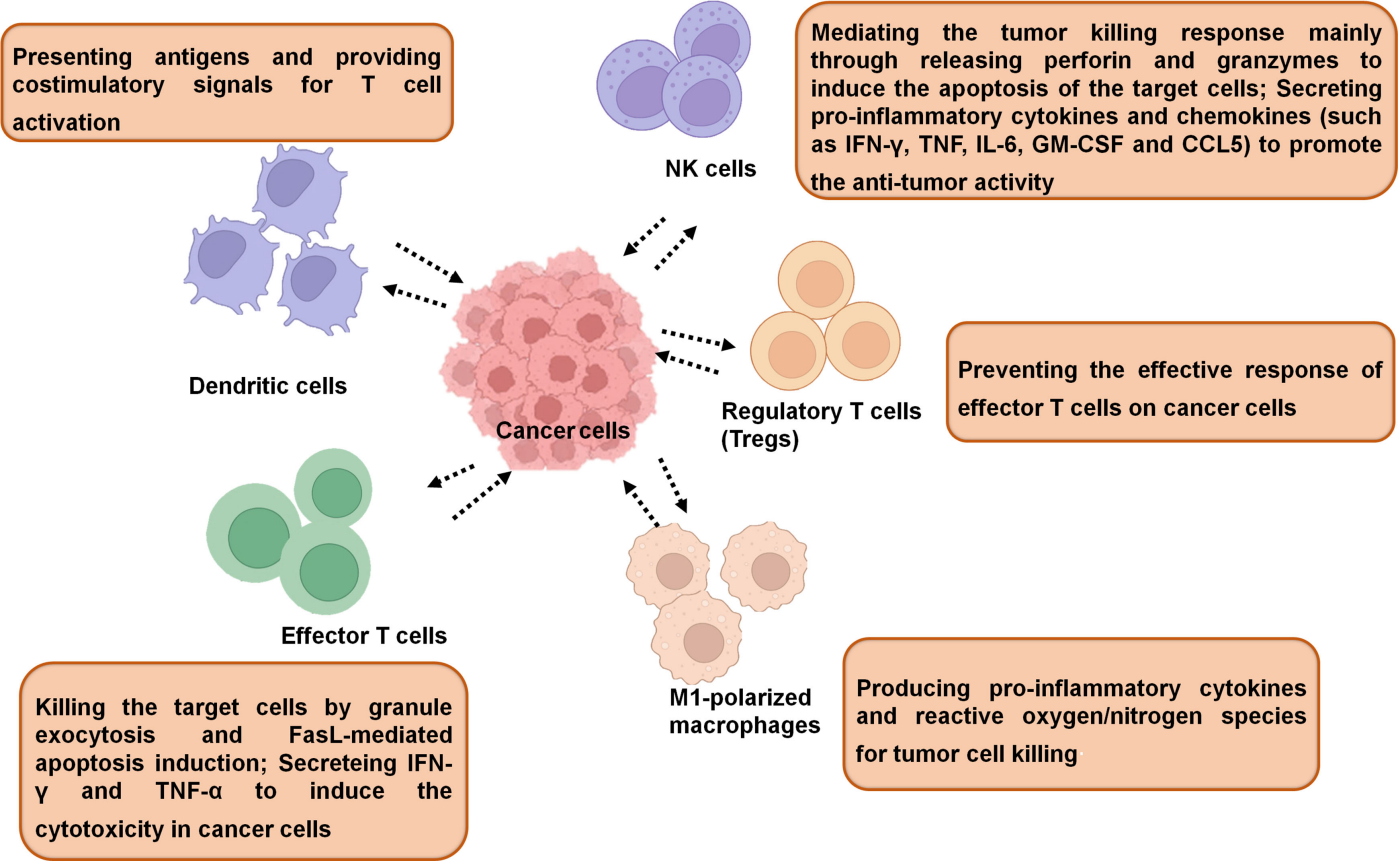
The immune cells in TME. The tumor-associated immune cells can be divided into two types: tumor-antagonizing and tumor-promoting immune cells.

**Figure 2:**
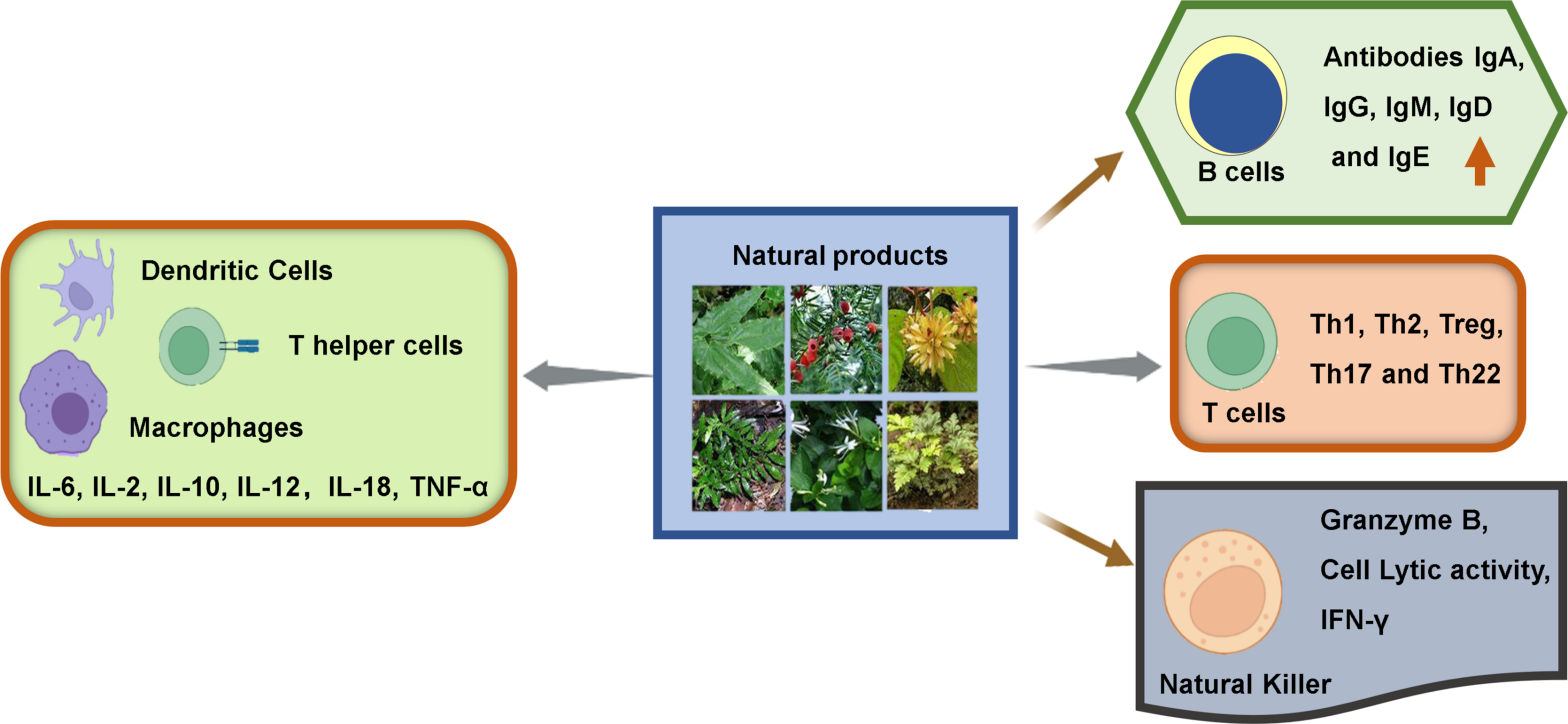
Immunomodulatory effects of natural products on immune cells within TME.

In general, the immune cells that have an effect on tumors can be divided into two types: tumor-promoting and tumor-antagonizing immune cells. These two types of cells play various roles at different stages of tumor progression (3, 19). The tumor-antagonizing immune cells mainly consist of NK cells, effector T cells (including effector CD4+ T cells and CD8+ cytotoxic T cells), M1-polarized macrophages and DCs. Except for the tumor-antagonizing immune cells, there are a plenty of tumor-promoting immune cells mainly consisting of regulatory T cells (Tregs) (4) (**Figure 1**). Natural products can activate specific intrinsic immune cells to kill tumor cells by enhancing antigen presentation or cellular immune processes. They can also inhibit the formation of blood vessels in the TME and the metastasis of tumors by suppressing certain intrinsic immune cells (**Figure 2**).

## Effects of natural products on monocytes and macrophages

Monocytes/macrophages are derived from bone marrow stem cells, which can enter the bloodstream and be distributed in various organ tissues after developing into monocytes.

In the process of participating in innate immunity, monocytes/macrophages can identify pathogen-associated molecular patterns (PAMP) and play an important role in innate immunity. On the other hand, monocytes/macrophages also can mediate and promote inflammatory responses and implement immunity killing, and antigen presentation (20, 21). Tumor associated macrophages (TAM) are traditionally classified into two kinds, termed as polarization: the immunosuppressive/anabolic M2 phenotype and the classical inflammatory M1 phenotype. M1 macrophages are the main effector cells for host destruction of pathogens and M2 macrophages have anti-inflammatory and angiogenic functions in tumors (22, 23). M1 can produce pro-inflammatory cytokines and reactive oxygen/nitrogen species, which are essential for host defense and tumor cell killing and are therefore considered to be ‘good’ macrophages (24).

**Figure 3:**
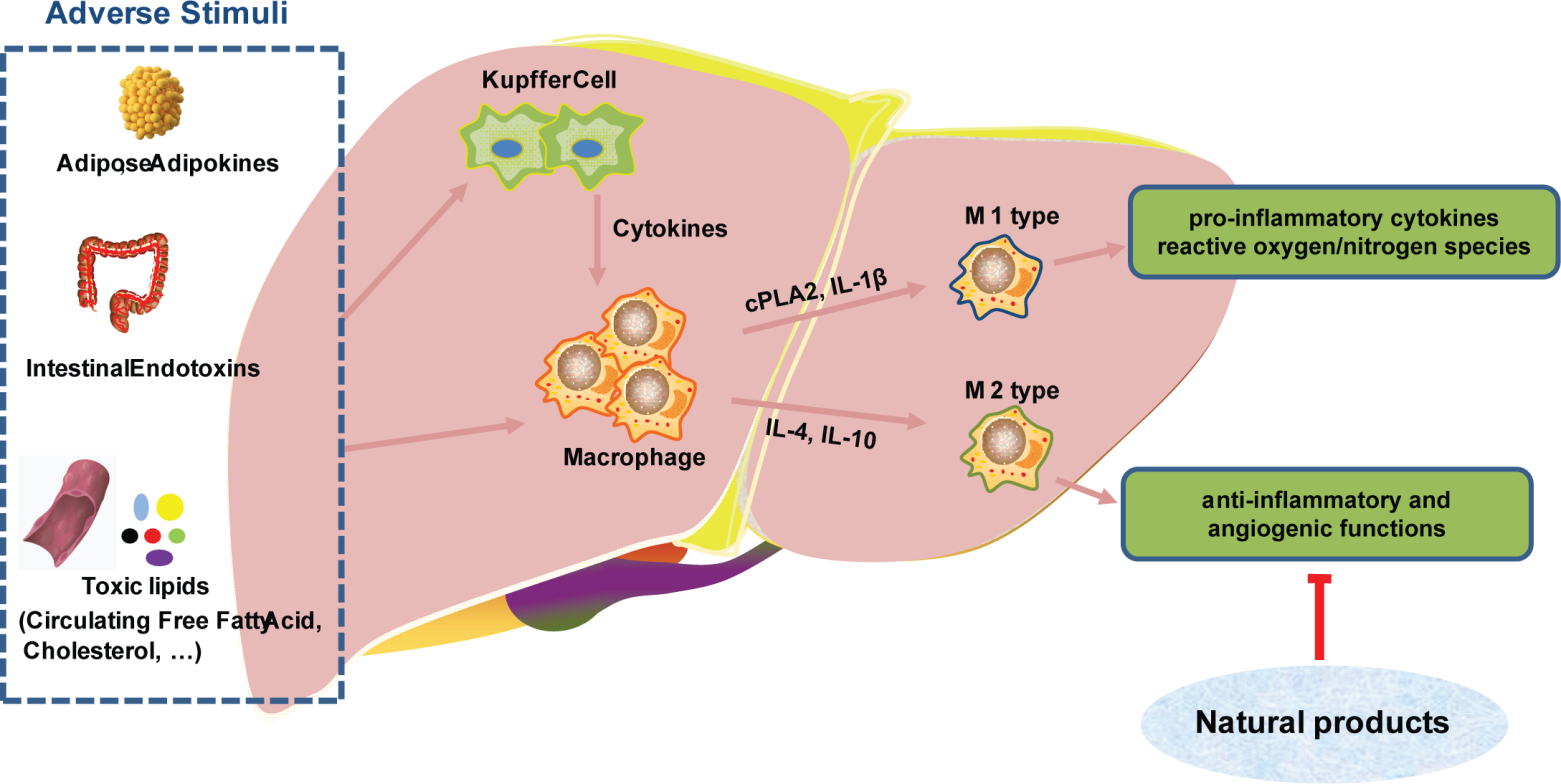
Natural products targeting macrophages in the treatment of liver cancer.

Natural products can inhibit the M2-like polarization of TAM and block tumor growth and migration. Emodin attenuated tumor growth by inhibiting IRF4, STAT6, and C/EBPβ signaling and M2-like polarization (25). Astragalus has been demonstrated to have anti-inflammatory and anti-fibrotic properties. Astragalus can inhibit the aggregation and activation of monocytes/macrophages, and reduce the production of TGF-β1 at the peritoneal site (26). Phenylpropanoid is the main active ingredient in ginger, which can directly inhibit cytoplasmic phospholipase 2 (cPLA2) and IL-1β in macrophage expression (27). Inonotus sanghuang, a medicinal plant rich in quercetin, isorhamnetin, quercitrin, rutin and chlorogenic acid, has been demonstrated to decrease inflammation *via* altering the interaction of macrophages and fat cells. It has been proposed that doing so may improve insulin resistance and the metabolic syndrome (28). Additionally, Garlic water-soluble extract has been shown to elevate intracellular thiol and glutathione concentrations in human primary monocytes. These results suggest that the extract of Garlic can regulate the differentiation of monocytes into macrophages, thereby playing a protective role (29). Macrophages are critical players in the development of nonalcoholic steatohepatitis (NASH) and hepatocarcinoma. Natural products have crucial roles in modulating macrophage activation, recruitment, and polarization, making them promising treatment possibilities for hepatocarcinoma (**Figure 3**) (30). Angiogenesis in the tumor microenvironment not only nourishes tumor cells and promotes their growth, but is also closely associated with tumor metastasis. Lentinan is a bioactive compound extracted from Lentinus edodes, which promotes the expression of the angiogenesis inhibitory factor IFN-γ, thereby inhibiting tumor angiogenesis (31).

## Effects of natural products on dendritic cells

DCs are the most powerful antigen-presenting cells, acting to condition the adaptive immune system to identify foreign antigens and serving as a link between the innate and adaptive immune responses (32). Different subsets of DCs can induce naïve CD4^+^ T cells to develop into Th1, Th2, Th17 and Treg cells, thereby modulating T cell-mediated immune response types that act as effector cells in the innate immune response (33, 34). DCs can exert their ability to induce, regulate and control T cell responses (35). Infiltration of mature, active DCs into the tumor increases immune activation and recruitment of immune effector cells.

Natural products can enhance anti-tumor immunity by promoting DCs maturation. Ganoderma lucidum polysaccharides, the main biologically active ingredient of Ganoderma lucidum, efficiently stimulate the activation and maturation of human monocyte-derived DCs. Ganoderma lucidum polysaccharides can also increase the expression of CD80, CD86, CD83, CD40, CD54, and human leukocyte antigen DR on the DC surface (36). Lycium barbarum polysaccharide (LBP), the main active ingredient in lycium barbarum, upregulates CD11c expression and induces DCs maturation through the TLR2/TLR4-mediated NF-κB pathway. LBP-treated DCs are more effective in promoting lymphocyte activation and proliferation, enhancing the immune response (37). Recently, there is increasing evidence that components of natural products can modulate the immune system by targeting DCs, including flavonoids, polysaccharides, phenolic compounds, saponins, and so on (38).

## Effects of natural products on natural killer cells

NK cells are also produced from bone marrow stem cells, which are mostly found in peripheral circulation and serve as the body’s first line of defense. At the same time, it can also participate in the cellular immune response and play an important role in tumor diseases *in vivo* (39). NK cells have a powerful cytolytic activity and play an important role in immune control (40). NK cells mediate the tumor killing response mainly by releasing perforin and granzyme to induce apoptosis in target cells (41). In addition, NK cells secrete chemokines and pro-inflammatory cytokines to promote anti-tumor activity (42). Besides, the NK cells may promote the formation and response of tumor-specific CD4+ and CD8+T cells (43).

The production of IL-2 and IFN- by CD4^+^ T cells can activate NK cells (44). When activated, NK cells release perforin and granzyme B, which cause apoptosis and necrosis in target cells. Numerous studies have revealed that resveratrol can activate and enhance the killing ability of NK cells. The low concentration of resveratrol can increase the expression of NKG2D and IFN-γ in NK cells (45). The resveratrol treatment group can upregulate its anti-tumor and anti-infective abilities by enhancing the activity of NK cells (46). In animal models, green tea catechin metabolitesincrease NK cell cytotoxicity, while quercetin increases NK cell lytic activity (47, 48). Berries high in flavonoids and pro-anthocyanidins not only prevent the progress of cancer but also play a role in the modulation of NK cell (49). After taking garlic extract for 90 days, NKG2D was found to be up-regulated in NK cells. It proves that garlic extract can improve the activity of NK cells and enhance immunity (50). When vitamin A, B, C, D, and E are applied to NK cells, they have stimulatory properties. Although the precise mechanism remains unknown in the majority of cases, components appear to be promising candidates for NK cell-stimulating drugs in tumors (51).

## Effects of natural products on regulatory T cells

Regulatory T (Treg) cells are a subtype of T cells with immunomodulatory functions that are closely related to the pathological processes of a variety of human tumors (52). When the suppressive function of Treg cells is compromised, the activity of helper T cells (Th) and killer T cells can induce autoimmune disorders, such as rheumatoid arthritis, multiple sclerosis, and systemic lupus erythematosus (53, 54). On the contrary, the immunosuppressive function of Treg cells is too strong to cause tumor immune escape, such as tumorigenesis (55, 56). Similar to M2 macrophages, Treg cells can inhibit the release of cytokines from Th1 cells and promote angiogenesis in the tumor microenvironment (57).

Kaempferol enhanced Treg cell immunosuppressive activity by inhibiting the activity of proto-oncogene serine/threonine kinase (PIM1) (58). The compound triptolide isolated from Triptolide obviously inhibited the Th2, Th1, and Th17 cell-mediated inflammatory responses and up-regulated the expression of FOXP3 (59). Evidence has demonstrated that lentinan decreased tumor vascular function in a non-T-cell dependent manner by increasing IFNγ production, and showed anti-tumor effect in LAP0297 lung tumor model (31). Lentinan significantly inhibited anti-inflammatory IL-10 and TGF-β 1, and increased the expression of pro-inflammatory chemokines/cytokines (IFN-γ and TNF-α) and IL-12, and decreased immunosuppressive Treg cells. The downregulation of Treg cells is associated with the over-induction of IFN-γ and TNF-α in non-small cell lung cancer (NSCLC), and the inflammatory state of Lentinoglycan treated NSCLC patients can change from Th2 to Th1 (60).

## Effects of natural products on effector T cells

Effector T cells include CD8+ cytotoxic T cells (CTLs) and effector CD4+ T cells. In the “activated” state, CTL can induce target cancer cells killing through granule cytokinesis and Fas ligand (FasL)-mediated apoptosis (61). CD4+ T cells are helper T cells that aid in the activation and regulation of immune cells. CD4+ T cells can directly help CD8+ T cell activation and proliferation (62–65). Furthermore, they can also help to shape CD8+ T cells into memory CTLs (66). When the T cell receptor (TCR) is activated, CD4+ T cells differentiate into Th1 or Th2 cells. The balance of Th1 and Th2 plays vital roles in the progress of cancers. With further study of T cells, researchers discovered a new T cell subpopulation called Th17 cells (67, 68). Th17 cells produce IL-17, IL-22, and chemokine ligands 20 (CCL20) (69, 70).

Ginsenosides are the active ingredients of ginseng. Studies have confirmed that ginsenoside Rg1 has a direct effect on the activity of Th and the development of the Th1/Th2 system (71). In addition, ginsenoside Rg1 can selectively enhance the expression of germline transcription products (GLTs), increase the production of IgA antibodies and promote humoral immunity (72). In a mouse model of melanoma, ginsenoside Rh2 induced a large number of CD4+ and CD8a+T lymphocytes to infiltrate into the tumor tissue, indicating an enhanced immune response and enhanced cytotoxicity of lymphocytes to melanoma cells B16-F10 (73). In mice, polyphenols, such as apigenin and chrysin inhibit ovalbumin immunization-induced serum IgE by downregulating Th2 responses (74). Likewise, tea polyphenols, such as EGCG, diminish Th1 differentiation and the numbers of Th17 and Th9 cells (61), while resveratrol reduces Th17 cell counts (75). Resveratrol dramatically reduced the fraction of CD4+CD25+ cells among CD4+ T cells in both *in vitro* and *in vivo* tests, demonstrating a dose-dependent mechanism (76).

## Natural products effectively expanding indications of various types cancer immunotherapy

Recent studies have shown that natural products can enhance the therapeutic effect of cancer vaccines and immune checkpoints inhibitors. Next, we focus on how natural products can improve both treatments through multi-cellular and multi-pathway modulation.

## Effects of natural products on the immune checkpoints inhibitors

Immune checkpoint molecules can regulate the immune state by activating or inhibiting immune signaling pathways. Overexpression of some of these molecules in TME leads to T cell dysfunction and ultimately promotes immune escape and tumor survival. Some immune checkpoint antibodies have been used in clinical anti-tumor therapy (77).

The PD-1/PD-L1 pathway, which promotes T cell functional failure, apoptosis and anergy, has stood out among immunological checkpoints due to the outstanding treatment outcomes in many studies (78). However, the presence of an immunosuppressive microenvironment in tumors limits the use of anti-PD-1/PD-L1 antibodies. Natural products have been reported to be key screening targets for PD-1/PD-L1 small molecule inhibitors and reversal of immunosuppression. Besides the ability to regulate the expression of PD-1 and PD-L1, the combination of natural products with anti-PD-1/PD-L1 antibodies has also shown excellent therapeutic efficacy.

The triterpenoid saponin isolated from Anemone flaccida inhibits the growth of hepatocellular carcinoma cells by downregulating the STAT3 signaling pathway to block the activation of PD-1 and PD-L1 (79). Ganoderma lucidum polysaccharide combined with paclitaxel (PTX) preserves the exhausted state of tumor-infiltrating lymphocytes (TILs) by downregulating PD-1 expression (80). Resveratrol can downregulate PD-L1 expression by activating HDAC3/p300/NF-κB signaling pathway in colorectal and breast cancer cells (81). Liu et al. find that combination of andrographolide isolated from Andrographis paniculata and anti-PD-1 antibody is more effective than monotherapy in the treatment of CT26 colon cancer (82). Diosgenin isolated from Acacia concinna in combination with anti-PD-1 antibody can effectively promote necrosis and apoptosis of melanoma cells. Furthermore, their findings suggest that the mechanism of diosgenin sensitivity to anti-PD-1 antibodies mainly contributes to the regulatory function of gut microbiota (83). Therefore, natural products have their unique advantages in immune checkpoints therapy.

## Effects of natural products on the cancer vaccines

Cancer vaccines, including cancer treatment vaccines and cancer prevention vaccines, are one of cancer-specific active immunotherapies. The effectiveness of cancer vaccines depends on the optimal combination of adjuvant, antigen, vaccination route and vector. Natural products can improve the immune-stimulate effect of cancer vaccines as adjuvants. QS-21 isolated from Quillaja Saponaria Molina can promote the antigen presentation process and remodel the immunosuppression by regulating Th1 cytokines. Meanwhile a series of Phase I-III clinical trials (leukemia, carcinoma,prostate, ovary, or lung) have investigated the effect of QS-21 as an immune adjuvant in cancer vaccines designed (84). Curcumin has the potential to improve the therapeutic outcome of Bacillus Calmette-Guerin and significantly enhance the efficacy of TRP2 peptide vaccine against melanoma. In addition, curcumin has been reported to inhibit IDO expression by blocking the JAK-STAT1 signaling pathway and to sensitize melanoma FAPαc vaccine by this mechanism (85).

In addition to the effective activation of CD8+ cytotoxic T lymphocytes, cancer vaccines must also face a challenge: poor immunogenicity. It is reported that natural products can enhance tumor immunogenicity by inducing immunogenic cell death (ICD) effects. The pathway of ICD-induced tumour cell death relies on damage-associated molecular patterns (DAMPs) such as heat shock proteins (HSPs), high mobility group box 1 (HMGB1) and calreticulin (CRT), making tumour cells a “therapeutic vaccine” that can induce anti-tumour immunity (86). Capsaicin has been reported to trigger ICD effects in primary effusion lymphoma (PEL) cells by inducing exposure to DAMPs (87). Ginsenoside Rg3 isolated from ginseng can induce ICD and enhance interferon g (IFN-g) secretion to inhibit tumor growth (88). oreover, shikonin can improve the expressions of MHC II and CD86, and enhance tumor-immunogenicity of tumor vaccines *via* ICD (89).

## Discussion

Given the multi-pharmacological activity and chemical diversity, natural products have been described as a non-substitutable source of clinical therapeutics for human tumors. Natural products can mediate multiple immune responses and reduce the tumor escape. They enhance the interaction of immune cells while decreasing the expression of pro-inflammatory cytokines. Previous studies have shown that natural products can exert anticancer activity through immune regulation *in vivo* and *in vitro*. Unlike traditional chemoimmunotherapy in tumor, natural products display many advantages, such as wide sources, less toxic and side effects, as well as diverse immunomodulatory activities, suggesting that natural products have an attractive prospect in the research of novel tumor immunotherapy. The combination of natural products and chemotherapeutic drugs may exert stronger therapeutic effect than chemotherapeutic drugs alone in tumors, which has synergistic and reducing toxic and side effects.

Although natural products have made encouraging appear promising and progress in various studies as modulators of tumor immunotherapy. For natural products to be better used in cancer immunotherapy, a number of issues still need to be addressed. First of all, natural products will face the problem of individual patient differences, TME differences, tumor heterogeneity. Secondly, a deeper and more comprehensive exploration of the signalling pathways of the immune system relevant to tumor immunotherapy is needed to help select more effective natural products. Thirdly, most natural products have a wide range of pharmacological effects, but their targets and molecular mechanisms relevant to tumour immunity have not been fully elucidated.

Above all, the emerging role of natural products in tumor immunotherapy still has greater potential and deserves attention. Future research can screen natural products for targeted antitumor immune drugs using advanced technologies, such as metabolomics, single-cell sequencing, novel drug delivery technologies, and computer-aided design techniques.

## Author contributions

X-QJ conceptualized the ideas. X-QJ, NL, W-JF, Z-HL, and J-LH performed the literature search, drafted the original manuscript, and drew the figures. Z-SL revised the manuscript. All the authors approved the final version of the manuscript.

## Funding

This work was financially supported by General Program of the National Natural Science Foundation of China Joint project (NO. U21A20348), National Natural Science Foundation of China General Project (No.81970633), Heilongjiang Provincial Higher Education Institutions Basic Research Business Expenses (2021KYYWF0046).

## Conflict of interest

The authors declare that the research was conducted in the absence of any commercial or financial relationships that could be construed as a potential conflict of interest.

## Publisher’s note

All claims expressed in this article are solely those of the authors and do not necessarily represent those of their affiliated organizations, or those of the publisher, the editors and the reviewers. Any product that may be evaluated in this article, or claim that may be made by its manufacturer, is not guaranteed or endorsed by the publisher.
